# Toxicological Profiling and Long-Term Effects of Bare, PEGylated- and Galacto-Oligosaccharide-Functionalized Mesoporous Silica Nanoparticles

**DOI:** 10.3390/ijms242216158

**Published:** 2023-11-10

**Authors:** Irene Barguilla, Vicente Candela-Noguera, Patrick Oliver, Balasubramanyam Annangi, Paula Díez, Elena Aznar, Ramón Martínez-Máñez, Ricard Marcos, Alba Hernández, María Dolores Marcos

**Affiliations:** 1 Grup de Mutagènesi, Departament de Genètica i de Microbiologia, Facultat de Biociències, Universitat Autònoma de Barcelona, 08193 Cerdanyola del Vallès, Spainricard.marcos@uab.cat (R.M.); 2 Instituto Interuniversitario de Investigación de Reconocimiento Molecular y Desarrollo Tecnológico, Universitat Politècnica de València, Universitat de València, 46022 Valencia, Spain; 3 Unidad Mixta UPV-CIPF de Investigación en Mecanismos de Enfermedades y Nanomedicina, Universitat Politècnica de València, Centro de Investigación Príncipe Felipe, 46012 Valencia, Spain; 4 Unidad Mixta de Investigación en Nanomedicina y Sensores, Instituto de Investigación Sanitaria La Fe (IISLAFE), Universitat Politècnica de València, 46026 Valencia, Spain; 5 CIBER de Bioingeniería, Biomateriales y Nanomedicina (CIBER-BBN), Instituto de Salud Carlos III, 28029 Madrid, Spain

**Keywords:** mesoporous silica nanoparticles, long-term effects, toxicological profiling

## Abstract

Mesoporous silica nanoparticles (MSNs) are amongst the most used nanoparticles in biomedicine. However, the potentially toxic effects of MSNs have not yet been fully evaluated, being a controversial matter in research. In this study, bare MSNs, PEGylated MSNs (MSNs-PEG), and galacto-oligosaccharide-functionalized MSNs (MSNs-GAL) are synthesized and characterized to assess their genotoxicity and transforming ability on human lung epithelial BEAS-2B cells in short- (48 h) and long-term (8 weeks) exposure scenarios. Initial short-term treatments show a dose-dependent increase in genotoxicity for MSNs-PEG-treated cells but not oxidative DNA damage for MSNs, MSNs-PEG, or for MSNs-GAL. In addition, after 8 weeks of continuous exposure, neither induced genotoxic nor oxidative DNA is observed. Nevertheless, long-term treatment with MSNs-PEG and MSNs-GAL, but not bare MSNs, induces cell transformation features, as evidenced by the cell’s enhanced ability to grow independently of anchorage, to migrate, and to invade. Further, the secretome from cells treated with MSNs and MSNs-GAL, but not MSNs-PEG, shows certain tumor-promoting abilities, increasing the number and size of HeLa cell colonies formed in the indirect soft-agar assay. These results show that MSNs, specifically the functionalized ones, provoke some measurable adverse effects linked to tumorigenesis. These effects are in the order of other nanomaterials, such as carbon nanotubes or cerium dioxide nanoparticles, but they are lower than those provoked by some approved drugs, such as doxorubicin or dexamethasone.

## 1. Introduction

The applications of nanomaterials are widely and rapidly growing. The interest in their use in biomedicine has increased greatly in the past years as such materials embody an attractive framework where new tools for imaging, diagnostic, and therapeutic purposes are flourishing [1]. Within this field, mesoporous silica nanoparticles (MSNs) are rising as some of the most promising and customizable drug delivery systems [2,3]. MSNs are formed from silicon dioxide arranged to obtain spherical particles with tunable pores from 2 to 50 nm, which can be loaded with selected cargos such as fluorophores, dyes, drugs, and enzymes or small peptides [4]. Moreover, MSNs can turn into stimuli-responsive delivery systems by capping the pores with different molecules that act as gatekeepers (also known as molecular gates) allowing cargo delivery only when specific stimuli are present [5,6]. Such systems have been used not only for drug delivery purposes [7,8,9,10] but also for chemical communication strategies [11] and sensing [12,13].

As MSNs gain popularity for potential biomedical applications, their biocompatibility has been often evaluated mostly analyzing different short- to mid-term endpoints. Generally, MSNs are described as biocompatible [3]. In fact, silica and its degradation products have been ‘generally recognized as safe’ (GRAS) by regulatory agencies such as the FDA and silica is used in cosmetics and in the food industry as an additive [14]. Nevertheless, the potential for MSNs to cause adverse effects in biomedical applications still remains controversial. In many in vitro and in vivo studies, MSNs revealed no cytotoxicity, including histopathological evaluation, even when relatively high concentrations were used [15,16].

Nonetheless, some studies found a correlation of MSN uptake and in vitro cytotoxicity, inducing ROS production which can damage cells and tissues, either inducing necrosis or apoptosis, inflammatory processes, and genotoxicity [17,18,19,20]. This cytotoxicity and ROS production have been suggested to be associated with silanol abundance on the surface of MSNs, which can disrupt the membrane components, the cytoskeleton, and nuclei components [21]. For instance, a recent in vitro finding demonstrated that bare MSNs could induce apoptosis, ROS, and DNA damage at high doses and repeated exposures in human corneal epithelial cells [22]. Another study described that MSNs were involved in intestinal damage in mice after sub-acute exposures, as well as ROS production, inflammasome activation, apoptosis, and loss of autophagy flux in intestinal epithelial cells [23]. Cardiac and pulmonary toxicity due to increased ROS levels and inflammation after MSN exposure have also been described [24].

Moreover, it has been widely reported that surface functionalization of MSNs can significantly increase, diminish, or even prevent adverse effects [25]. In fact, functionalization of MSNs greatly affects the chemical properties of the surface, such as the net surface charge and polarity, which influences the interactions with cells, the adsorption of proteins, and the nanoparticles’ stability [26]. For example, no toxicity was found for MSN functionalized with folate in cell culture models [27,28]. Another study analyzed the acute toxicity of MSNs in immune-competent mice, establishing the highest maximum tolerated dose of amino-functionalized MSNs at 100–150 mg/kg and non-functionalized MSNs at 30–65 mg/kg [29], suggesting that they have a low toxicity. In addition, nanoparticles’ morphology can also affect some cell functions such as apoptosis, adhesion, migration, and proliferation, which are related to cytoskeleton organization [30].

The contradictory results found in the literature in relation to the toxicity of MSNs bring out the necessity to carry out additional thorough biocompatibility studies. These inconsistent conclusions are evidenced even more in the long-term effects of MSNs. While some evaluations did not find acute or chronic toxicity or inflammation in vivo after treatment with MSNs for a month [31] or one year [32], other authors claimed that the low degradability of MSNs in vivo could lead to long-term toxicity in various tissues or cause diseases [33,34]. Another study with in vivo models reported the toxicity of submicron MSNs with acute exposure (10 days), but this was considerably alleviated with chronic exposure (180 days) [35]. Nonetheless, there is a remarkable lack of studies in which the long-term effects of MSN exposure are analyzed.

Based on the above, and to advance our understanding of MSN toxicity, we report herein in vitro short- and long-term toxicity and genotoxicity studies on human bronchial epithelial cells (BEAS-2B) using three different MSNs: (i) bare MSNs (MSNs), (ii) PEGylated MSNs (MSNs-PEG), and (iii) galacto-oligosaccharide-functionalized MSNs (MSNs-GAL). MSNs-PEG [36] and MSNs-GAL [37] have already been used as drug delivery systems in in vitro and in vivo studies.

## 2. Results

### 2.1. Synthesis and Characterization of Materials

Bare MSNs, MSNs-PEG and MSNs-GAL were synthetized following the protocols described in the experimental section and were characterized using standard techniques. Powder X-ray diffraction (PXRD) patterns of the bare MSNs (as-made and calcined) are shown in Supplementary Figure S1A. The PXRD patterns display a typical MCM-41 hexagonal arrangement of pores, whose peaks can be indexed as (100), (110), (200), and (210) Bragg reflections. In addition, it is observable that there is a right drift of peaks in calcined MSNs compared to as-made MSNs. This fact can be attributed to an approximate cell contraction produced by the reorganization of the mesoporous structure and further condensation of the silanol groups due to the surfactant removal process in the calcination process.

N_2_ adsorption−desorption isotherms of bare MSNs were recorded (Supplementary Figure S1B), in which a typical curve for MCM-41-like mesoporous solids was obtained, corresponding to a type IV isotherm. This indicates nitrogen condensation inside the mesopores by capillarity with an acute adsorption step at intermediate P/P_0_ values (0.3–0.4). Moreover, the minimum hysteresis loop confirms the proper formation of uniform cylindrical mesopores, in which nitrogen freely adsorbs and desorbs. Using the adsorption curve and the Barrett-Joyner-Halenda (BJH) model, a narrow pore distribution centered at 2.88 nm and a pore volume of 0.944 cm^3^ g^−1^ were calculated. Furthermore, applying the Brunauer-Emmett-Teller (BET) model resulted in a value of 1118.6 m^2^ g^−1^ for the relative surface area of the calcined MSNs. In addition to mentioned data, a second feature can also be observed at high relative pressures (P/P_0_ > 0.9), corresponding to the filling of the interparticle space. Regarding the gated MSNs, the isotherms showed a reduction in capillary condensation because the pores were filled by dyes. The pore volume and surface area were reduced to ca. 0.1 cm^3^ g^−1^ and ca. 150 m^2^ g^−1^, respectively, which was in agreement with reported data for MSNs-PEG and MSNs-GAL [36,37].

The mesoporous structure of bare MSNs, MSNs-PEG and MSNs-GAL was also confirmed by transmission electron microscopy (TEM) analysis. Representative images in Supplementary Figure S2 confirmed that the solids were obtained as spherical particles with a diameter of around 82 nm ± 15 nm. Moreover, the MCM-41-like hexagonal structure of the mesopores can also be observed in the samples as pores and channels. Furthermore, the hydrodynamic diameters of the MSNs, MSNs-PEG, and MSNs-GAL were determined by dynamic light scattering (DLS) (Supplementary Figure S3). The size distribution of the hydrodynamic diameter obtained for MSNs was centered at 148 nm, whereas that found for MSNs-PEG and MSNs-GAL were centered at 245 nm and 300 nm, respectively. These results agree with the functionalization of the MSNs with PEG and GAL, which forms an organic layer around the nanoparticle increasing the hydrodynamic diameter of the material. The surface charge of the nanoparticles was monitored measuring their ζ potential, whose results were −37.1 mV for bare MSNs, −26.4 mV for MSNs-PEG, and −12.3 mV for MSNs-GAL. The negative surface charge of MSNs can be explained due to the presence of silanol groups on their surface, which can be deprotonated and form silanolate anions. When nanoparticles are functionalized, their negative charge decreases due to two processes: the reaction of silanol groups with alkoxysilanes and the shielding of the charge of free silanol groups by the anchored oligomers, i.e., PEG and GAL, which have no charge. The characterization results are summarized in Table 1.

The organic contents in the solids were also determined by thermogravimetric studies (Supplementary Figure S4). As summarized in Table 1, thermogravimetric data show that organic content increases with cargo loading and functionalization. Considering that the loss of mass in MSNs due to silanol condensation is ca. 3.5%, the organic contents are 23.1% and 18.2% for MSNs-PEG and MSNs-GAL, respectively.

Nanoparticles MSNs-PEG and MSNs-GAL were loaded with cargo (SafO and RhoB, correspondingly) to demonstrate the correct capping ability of the PEG and GAL functionalization. The controlled release performance of the gated materials was evaluated following the methodology described in the Supplementary Information (release assays). The release assays were monitored for 24 h. Both MSNs-PEG and MSNs-GAL showed a specific release of their cargo just in the presence of the corresponding stimulus (GSH and βGAL, respectively) and a negligible release in absence of them (Supplementary Figure S5).

### 2.2. Acute MSNs Exposure Does Not Lead to Toxic Effects in BEAS-2B Cells

**Figure 1:**
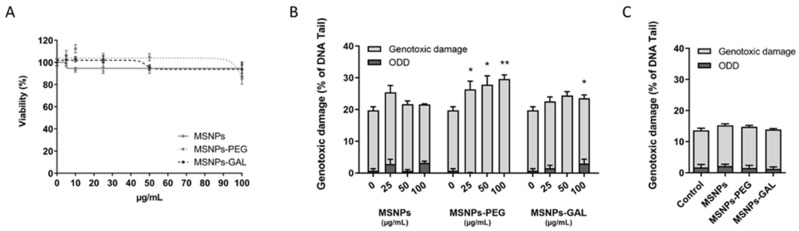
Cytotoxicity and genotoxicity evaluation of bare and gated MSNs on BEAS-2B cells. (**A**) Cell viability 48 h after the treatment with different doses of MSNs, MSNs-PEG, and MSNs-GAL. (**B**) Genotoxic and oxidative DNA damage induced by MSNs, MSNs-PEG, and MSNs-GAL at different concentrations after 48 h of short-term treatment. (**C**) Long-term genotoxic and oxidative DNA damage induced by MSNs, MSNs-PEG, and MSNs-GAL at 10 µg/mL concentration after 8 weeks of continuous exposure. Data represented as mean ± SEM (*n* = 3), one-way ANOVA with Dunnett’s post-test for exposed vs. unexposed or passage-matched controls. ** *p* < 0.01, * *p* < 0.05.

The analysis of cell viability after a 48 h short-term exposure to bare MSNs, MSNs-PEG, and MSNs-GAL shows a survival rate over 80% for the selected doses, ranging from 5 to 100 µg/mL. No differential effect of the nanoparticles’ coating was observed (Figure 1A). Therefore, a non-cytotoxic dose of 10 µg/mL was selected for the three solids to continue forward with a chronic BEAS-2B exposure.

### 2.3. MSNs Can Induce Slight Genotoxic Damage with Short-Term Exposure While This Effect Is Not Observed in Long-Term Exposed Cells

The genotoxic damage when cells are treated with nanoparticles is shown in Figure 1B for acute exposure (48 h at high doses) and in Figure 1C for chronic exposure (8 weeks). Regarding the acute exposure, neither MSNs nor the coated particles induce any oxidative DNA damage (ODD), with the exception of MSNs-GAL (100 µg/mL) where a slight increase was observed. In addition, bare particles do not lead to genotoxic damage, and there is barely any in the case of MSNs-GAL-exposed cells. Nevertheless, we have found that there is a slight dose-dependent increase in MSNs-PEG-induced genotoxicity. On the other hand, after 8 weeks of chronic exposure, no genotoxic or ODD was observed neither for the non-capped nor the gated MSNs, suggesting that cells adapt to the presence of low doses of MSNs.

### 2.4. Gated MSNs Show a Distinctive Transforming Potential after Chronic Exposure

To evaluate the effects of a longer exposure to the MSNs and assess the potential transforming ability of these particles, different transformation biomarkers were evaluated after 8 weeks of chronic exposure.

**Figure 2:**
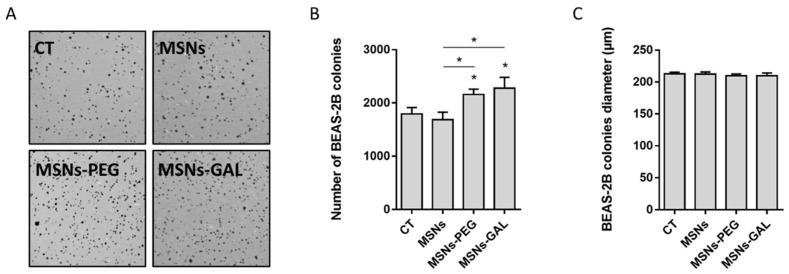
Long-term exposure to gated MSNs increases the cells’ colony-forming ability. The representative images (**A**) and bar graphs showing the colony number (**B**) and the colony size (**C**) of BEAS-2B cells treated with MSNs, MSNs-PEG, and MSNs-GAL compared to untreated controls after 8 weeks of long-term exposure. (**C**) Data represented as mean ± SEM (*n* = 3), one-way ANOVA with Dunnett’s post-test for exposed vs. passage-matched controls. * *p* < 0.05.

No remarkable changes were observed in the proliferation rate of the exposed BEAS-2B cells. Nevertheless, the anchorage-independent growth analysis in soft agar showed a gentle increased ability to form colonies for the MSN-PEG- and MSN-GAL-exposed cells when compared with the non-exposed controls and those exposed to bare MSNs (Figure 2A,B). No further effects in the colony size were observed (Figure 2C).

**Figure 3:**
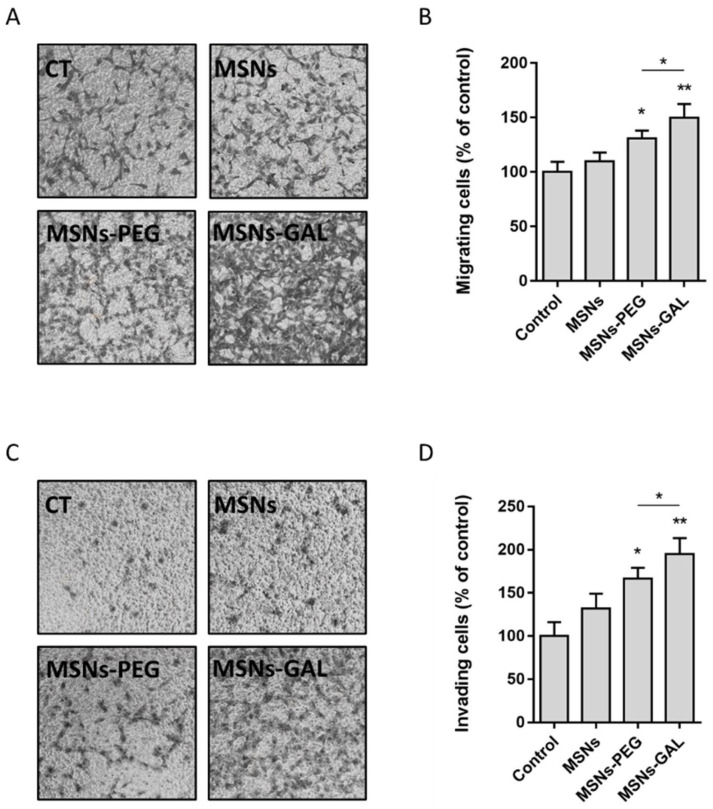
The migration and invasion potential of BEAS-2B cells increases when chronically exposed to gated MSNs. The representative images (**A**) and histogram (**B**) depicting the proportion of BEAS-2B cells able to migrate after continuous exposure to MSNs, MSNs-PEG, and MSN-GAL compared to passage-matched control for 8 weeks. The representative images (**C**) and histogram (**D**) revealing the invasion capability of BEAS-2B cells induced by MSNs, MSNs-PEG, and MSN-GAL in comparison to passage-matched control at 8 weeks. Data represented as mean ± SEM (*n* = 3), one-way ANOVA with Dunnett’s post-test for exposed vs. passage-matched controls. ** *p* < 0.01, * *p* < 0.05.

The evaluation of the migration (Figure 3A,B) and invasion (Figure 3C,D) potential of the exposed cells consistently showed that the cells chronically exposed to PEG- or GAL-coated-MSNs were more able to cross the transwell when compared both to bare MSNs-exposed cells and non-exposed controls. The increase in the migrating cells is ca. 30% and 65% when they are treated with MSNs-PEG and MSNs-GAL, respectively. In the case of the invading cells, the increment is ca. 50% and 90% for MSNs-PEG and MSNs-GAL, respectively.

### 2.5. Chronically Exposed Cells Undergo Secretome Changes Leading to Cell Growth Promotion

**Figure 4:**
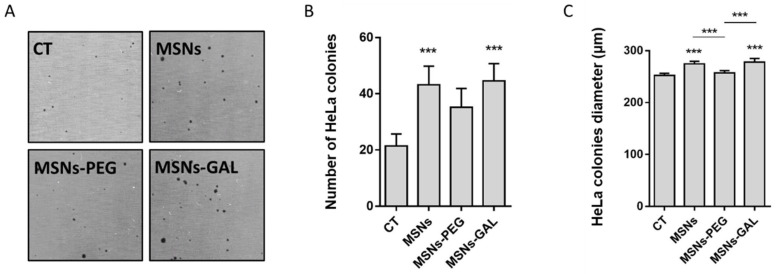
Enhanced number and size of colonies formed by HeLa cells in conditioned media or secretome from BEAS-2B cells treated with MSNs, MSNs-PEG, and MSN-GAL for 8 weeks by indirect soft agar. The representative images (**A**) and bar graph (**B**) showing the number of colonies formation by HeLa cells cultured in conditioned media from BEAS-2B cells exposed to MSNs, MSNs-PEG, and MSN-GAL vs. HeLa cells grown in conditioned media from untreated BEAS-2B cells at 8 weeks. The histogram (**C**) revealing the size of colonies by HeLa cells grown in conditioned media from BEAS-2B cells treated with MSNs, MSNs-PEG, and MSN-GAL vs. HeLa cells grown in conditioned media from untreated BEAS-2B cells at 8 weeks. Data represented as mean ± SEM (*n* = 3), one-way ANOVA with Dunnett’s post-test for exposed vs. unexposed or passage-matched controls. *** *p* < 0.001.

Cells chronically exposed to contaminants or other agents tend to undergo changes in their secretome. The indirect soft-agar assay evidenced that the secretome from MSNs and MSNs-GAL-exposed BEAS-2B cells was able to increase the colony growth of the already well-established tumoral HeLa cells (Figure 4A,B), and the colonies’ diameter slightly increases in these cases (Figure 4C).

## 3. Discussion

The advancement and success of the biomedical field largely depend on addressing the potential challenges it faces through innovation and technology. Hence, engineered nanoparticles are regarded as one of the most promising tools which could provide necessary answers in biomedicine or biomedical engineering [38]. Notably, MSNs are considered excellent candidates for biomedical applications because they possess unique physicochemical properties such as large surface area, enhanced drug adsorption, tunable pore size, and expected limited toxicity. Moreover, the possibility to control the cargo release by introducing surface modifications is a great asset in applications such as targeted drug delivery or bio-imaging [39,40]. Nevertheless, there is no consensus about the adverse effects the MSNs can provoke, remaining a controversial issue, in both acute (short-term) and chronic (long-term) exposure scenarios.

Although different approaches have been used to evaluate the DNA damage induced by engineered nanoparticles, including MSNs, the comet assay we have used in our study has been regarded as a sensitive tool to detect genotoxic and oxidative DNA damage in single cells, and it is amenable to the high-throughput screening of several engineered nanoparticles at once [41,42]. Chronic exposure (8 weeks) did not induce observable DNA damage, whereas for acute exposure (48 h at high doses), our results demonstrated slight dose- and functionalization-dependent genotoxic DNA damage, only being significant in the case of MSNs-PEG. Some studies performed to evaluate the acute exposure reported that bare MSNs were able to cause double-strand DNA breaks in human colon cancer (HT29) and in chicken bursa B lymphocyte (DT40) cells [17,43]. Similarly, MSNs were found to be genotoxic in human embryonic kidney cells as they cause genomic DNA degradation [44]. In contrast, other authors described that unmodified MSNs, aminated MSNs, and phosphonate MSNs at high concentrations and at exposures lasting for 12 and 24 h did not cause genotoxic DNA damage, possibly due to a lack of oxidative damage induction in rat pheochromocytoma PC12 cells [45].

To further characterize the long-term effects of the three MSN systems, we selected a battery of assays to evaluate their carcinogenic potential based on previous studies [46]. The anchorage-independent cell growth is regarded as one of the hallmarks of cancer development and can be estimated by using the soft-agar colony formation assay. This is a widely accepted assay to characterize the malignant cells which could proliferate and grow without adherence to a substrate [47,48]. Our results show a slight increase in the colony formation ability of cells exposed to MSNs-PEG and MSNs-GAL but a modest decrease in the case of bare MSN. Thus, it can be attributed to a mild transformation ability of gated MSNs. In addition, this transformation behavior was also observed for the secretome of chronically exposed cells, either to bare or gated MSNs. The cell transforming potential of MSNs was also suggested in short-term exposures (6 and 24h), where both colony formation and migration of CHO-K1 cells were observed [49]. Nonetheless, it should be indicated that the effects observed in long-term exposure to other nanoparticles, such as cerium dioxide nanoparticles [50], are much higher than those reported in this study. Even MSNs’ transformation ability is significantly lower than some approved drugs such as dexamethasone [51] or vinblastine [52]. Considering this, MSNs might be used as drug carriers with a reduced risk.

Likewise, cell migration and invasion assays using transwell or Boyden chambers are highly efficient and well-established methods for evaluating the metastasis of cancer cells [53]. Concordantly, these assays have previously been used to analyze the metastatic potential of cells triggered by the exposure to engineered nanoparticles. With this methodology, we have observed that MSNs-PEG and MSNs-GAL significantly induce the migrating and invading potential of cells chronically exposed to these nanoparticles. This increase is in the range of other studied nanomaterials. For example, He et al. [54] found increased metastasis of the CNT-transformed cells due to overexpression of mesothelin. Moreover, C60, single-walled CNTs, and graphene oxide nanoparticles were able to induce cell migration and invasion via the P2 × 7R-HMGB1-RAGE pathway [55]. However, promotion of cell migration and invasion is significantly higher (3–4-fold) in the case of some approved drugs with carcinogenic effects, such as doxorubicin [56,57]. Hence, the encapsulation of these kinds of drugs, such as doxorubicin, in MSNs could even mitigate their carcinogenicity and metastatic ability.

MSNs and their possible adverse effects must be evaluated, both in the short term and long term, for their effective translation to clinics [58]. In this sense, although exposure to MSNs is supposed to be short if applied medically, some authors point out the persistence of MSNs in the kidney, spleen, and lung in mice after intravenous administration [59]. In contrast, other studies state that the excretion of MSNs from the organism is rapid. For example, some authors determined that more than 90% of administered nanoparticles functionalized with fluorescein and phosphonate groups, and loaded with camptothecin, were excreted within 4 days [31].

The long-term exposure of MSNs in the tissues is currently an issue under discussion. Some studies described that different scaffolds of porous silica are degraded in oligomers and, in turn, orthosilicic acid [15]. This metabolite is water-soluble and finally excreted mainly through renal clearance [60], even in the range of four weeks, or also through hepatobiliary clearance [61]. In this elimination of the MSNs, the reticuloendothelial system (RES) plays a central role [21], based on the macrophage’s phagocytosis and degradation of MSNs into soluble products. Thus, the immune system, which is not simulated in the in vitro cell culture, is involved in the excretion of nanoparticles. In either case, our strategy of using low doses and chronic exposure was aimed to overcome the potential gaps for carcinogenic risk assessment of MSNs under in vitro conditions. It must be emphasized that one of the main limitations while assessing the genotoxic or carcinogenic potential of engineered nanoparticles could be that the short-term treatment of cells with high doses results in transient effects [62]. With the long-term exposure approach, it is possible to evaluate ‘end stage’ effects which require the cells to be repeatedly exposed to lower or sub-toxic doses of nanoparticles for several weeks.

## 4. Materials and Methods

### 4.1. Chemicals

Tetraethylorthosilicate (TEOS), 1-hexadecyltrimethylammonium bromide (CTAB), sodium hydroxide (NaOH), Rhodamine B (RhoB), Safranin O (SafO), (3-aminopropyl)triethoxysilane (APTES), (3-mercaptopropyl)trimethoxysilane (MPTMS), 2,2′-dipyridyl disulphide, poly(ethylene glycol)methyl ether thiol Mn 800 (PEG-SH), L-glutathione reduced (GSH), and β-galactosidase from Aspergillus oryzae (βGAL) were purchased from Sigma-Aldrich. Galactan from potato (GAL) was purchased from Carbosynth. Acetonitrile was purchased from Scharlab (Barcelona, Spain). All reagents and solvents were used as received without further purification.

### 4.2. General Techniques

Transmission electron microscopy (TEM), N_2_ adsorption–desorption isotherms, powder X-ray diffraction (PXRD), thermogravimetric analysis (TGA), elemental analysis (EA), and dynamic light scattering (DLS) were used to characterize the prepared materials. Instruments and protocols used are listed below. A JEOL JEM-1010 microscope was used to obtain representative TEM. Tristar II Plus equipment from Micromeritics was employed to record N_2_ adsorption–desorption isotherms. Samples were degassed at 120 °C in a vacuum overnight. The specific surface areas were calculated from the adsorption data within the low-pressure range using the BET (Brunauer–Emmett–Teller) model. Pore size was determined following the BJH (Barrett–Joyner–Halenda) method. A Bruker D8 Advance diffractometer (Cu Kα radiation) was used for PXRD measurements. A TGA/SDTA 851e Mettler Toledo balance was used for thermogravimetric assays. The protocol used an oxidant atmosphere, with a heating program that consisted of a heating ramp of 10 °C/min to 1000 °C, applying an isotherm at 100 °C over 60 min. Particle size and ƺ potential in solution were measured by a ZetaSizer Nano ZS (Malvern Instruments Ltd., Worcestershire, UK) equipped with a laser of 633 nm and the signal was collected at 173°.

### 4.3. Synthesis of MSNs

An amount of 1 g of CTAB was dissolved in 480 mL of deionized water, the temperature was adjusted to 50 °C, and the solution was stirred with a magnetic stir bar. Then, 0.28 g of NaOH dissolved in 3.5 mL of deionized water was added to the CTAB solution and the temperature was set at 80 °C. Once the temperature was reached, 5 mL of TEOS was added drop by drop and stirred vigorously for 2 h. A white precipitate was observed. The dissolution was left to cool. The solid product was centrifuged and washed with deionized water to neutralize the pH and dried in an oven at 60 °C (as-made MSNs). Finally, as-made MSNs were calcined at 550 °C for 5 h using an oxidant atmosphere (bare MSNs).

### 4.4. Synthesis of MSNs-PEG

A total of 200 mg of calcined MSNs and 56 mg of SafO were suspended in 10 mL of distilled water. The mixture was stirred for 24 h at room temperature, after which it was centrifuged once and dried at 60 °C. Then, 100 mg of the obtained solid was resuspended in 5 mL of acetonitrile. Shortly, 185 μL of MPTMS was added and the mixture was stirred for 5.5 h at room temperature, and then 220 mg of 2,2′-dipyridyl disulfide was added. The suspension was stirred for 12 h at room temperature and the resulting solid was filtered off and dried under vacuum. After that, 50 mg of this prepared solid was resuspended in 3 mL of acetonitrile, and 120 mg of PEG-SH was added. The mixture was stirred for 12 h. Afterwards, it was centrifuged and washed with abundant water to remove the excess SafO. The final solid, MSNs-PEG, was dried at 60 °C.

### 4.5. Synthesis of MSNs-GAL

Calcined MSNs (200 mg) and 76 mg of RhoB were suspended in 5 mL of acetonitrile. The mixture was stirred for 24 h at room temperature, after which 280 μL of APTES was added and the mixture was stirred for 5.5 h at room temperature. The resulting solid was filtered off and dried under vacuum. After that, 50 mg of this prepared solid was resuspended in 3 mL of distilled water and mixed with 95.75 mg of GAL dissolved in 3 mL of distilled water. The mixture was stirred for 21 h at room temperature. Afterwards, the suspension was centrifuged and washed with abundant water to remove the excess RhoB. The final solid, MSNs-GAL, was dried at 37 °C.

### 4.6. Cell Culture Conditions

BEAS-2B lung epithelial cells were grown in Dulbecco Modified Eagle Medium (DMEM) (Gibco, Paisley, UK) supplemented with 10% fetal bovine serum (FBS, Biowest, Nuaillé, France), 1% non-essential amino acids (NEEA, PAA), and 2.5 µg/mL Plasmocin (InvivoGen, San Diego, CA, USA) in a humidified atmosphere of 5% CO2 and 95% air at 37 °C.

### 4.7. MSNs Dispersion and In Vitro Long-Term Exposure

A dispersion of the different MSNs was prepared previously to the cell’s exposure. Briefly, MSNs were pre-wetted in 0.5% absolute ethanol and dispersed to a final concentration of 2.56 mg/mL in 0.05% bovine serum albumin (BSA) in double-distilled water. An 8-week exposure to 10 µg/mL MSNs was carried out in triplicate for each MSN coating. Passage-matched controls were maintained for comparisons.

### 4.8. Cytotoxicity Assay

To evaluate the effect of the different MSNs on cell survival, cell viability was determined by the Beckman counter method with a ZTM Series coulter-counter (Beckman coulter Inc., Brea, CA, USA). The day before the exposure, 80,000 cells were seeded on 12-well plates. Then, the cells were treated with increasing doses of MSNs (5, 10, 20, 50, 100 µg/mL). After 48 h of treatment, the cells were counted to assess cell viability.

### 4.9. The Comet Assay

The alkaline comet assay including the use of formamidopyrimidine DNA glycosylase (FPG) was performed as previously described [63] to determine the genotoxic and oxidative DNA damage in BEAS-2B cells induced by the 48 h or 8-week exposure to MSNs. Briefly, cells were collected by trypsinization, centrifuged, and resuspended in cold PBS at 17,500 cells/25 μL before mixing them with 0.75% LMP agarose at 37 °C (1:10) and dropping 7 μL of the mixture onto Gelbond^®^ (GF) sheet films. Two identical films were prepared and processed simultaneously in each experiment. GFs were immersed overnight in ice-cold lysis buffer at 4 °C (2.5 M NaCl, 0.1 M Na_2_EDTA, 0.1M Tris Base, 1% Triton X-100, 1% lauryl sarcosinate, 10% DMSO; pH 10) to allow for cell lysis. The day after, the GFs were washed twice (1 for 5 min, 1 for 50 min) in enzyme buffer at pH 8 (10 mM HEPES, 0.1 M KCl, 0.5 mM EDTA, 0.2 mg/mL BSA) at 4 °C, and then one of the replicates was incubated for 30 min at 37 °C in enzyme buffer and the other in FPG-containing enzyme buffer. Then, the GFs were washed with electrophoresis buffer (0.3 M NaOH and 1 mM Na_2_EDTA; pH 13.2) and placed into a horizontal gel electrophoresis tank where DNA was allowed to unwind for 35 min before initiating the electrophoresis, which was carried out for 20 min at 0.8 V/cm and 300 mA at 4 °C. The GFs were rinsed with cold PBS for 15 min before the fixation step, performed by immersing them in absolute ethanol for 2 h. GFs were air-dried overnight at room temperature and then stained for 20 min with SYBR Gold 1/10,000 in TE buffer (10 mM Tris, 1 mM EDTA pH 7.5). Finally, GFs were mounted and visualized with an epifluorescent microscope at 20x magnification. One hundred randomly selected comet images were analyzed by sample according to the percentage of DNA in the tail as scored with the Komet 5.5 Image analysis system (Kinetic Imaging Ltd., Liverpool, UK).

### 4.10. The Soft-Agar Assay

The ability of the 8-week exposed BEAS-2B cells to grow independently of anchorage was determined by the colony formation in soft agar. To this aim, BEAS-2B cells were collected and filtered through a 40 µm mesh, obtaining single-cell suspensions. Subsequently, 65,000 cells resuspended in 1.75 mL of DMEM containing 10% FBS and 2.5 µg/mL plasmocin were mixed in a 1:1:1 ratio with 2X DMEM, containing 20% FBS, 2% NEEA, 2% L-Glu 200 mM, and 2% penicillin-streptomycin, and with 1.2% of bacto-agar (DIFCO, MD, USA). With this mixture, triplicates of 20,000 cells each were prepared by dispensing 1.5 mL over a 0.6% base agar (supplemented with 2x DMEM) in each well of a 6-well plate. After 21 days of incubation at 5% CO_2_, 95% air, and 37 °C, the cell colonies were stained in a 24 h incubation with 1 mg/mL of (2-p-iodophenyl)-3-3(p-nitrophenyl)-5-phenyl tetrazolium chloride (INT; Sigma, MO, USA). Then, the plates were scanned, and the colonies were counted using the colony cell counter enumerator software OpenCFU (3.9.0).

To assess the tumor-promoting ability of long-term exposed cells, we performed the indirect soft-agar assay. In this modified version of the protocol, the 72 h conditioned media of the treated BEAS-2B cells were collected. Cells with a known anchorage-independent growth capability such as HeLa cells were passed through a 40 µm mesh to obtain single-cell suspensions. Then, 35,000 cells were suspended in 1.75 mL of BEAS-2B cells CM and mixed in a 1:1:1 ratio with 2X DMEM and 1.2% bacto-agar to prepare triplicates of 10,000 cells each. The remaining steps were performed as indicated previously.

### 4.11. Migration and Invasion Assay

To assess the aggressive features of the MSN-transformed cells, direct migration and invasion assays were performed. To carry out the invasion assay, chronically exposed BEAS-2B cells at 80% confluence were deprived of FBS for 24 h before the assay. The day of the assay, a 180 µL 1:2 dilution of Matrigel^®^ (Costar-Corning, Corning, NY, USA) in FBS-free DMEM:F12 with 0.1% BSA was used to coat each 8 µm pore size polycarbonate membrane 24 mm transwell insert (Costar-Corning, Corning, NY, USA). The Matrigel^®^ mixture was left to sit and dry for 1 h in the cell incubator at 37 °C. The bottom chamber of the transwell insert was filled with 2.5 mL DMEM complemented with 15% FBS as the chemoattractant medium. A single-cell suspension containing 600,000 FBS-deprived cells in 1.5 mL of FBS-free DMEM with 0.1% BSA was added on top of the transwell Matrigel^®^-coated membrane. Cells were then allowed to invade for 48 h at 37 °C. Invading cells in the basal part of the transwell were stained with Crystal violet (Sigma-Aldrich, Darmstadt, Germany) and photographed for quantification using Image J.

To evaluate cell migration, an alternative version of the protocol following the steps described above was carried out where the cells were seeded on the top of the transwell without the Matrigel^®^ coating.

### 4.12. Statistics

The unpaired Student’s *t* test or one-way ANOVA followed by Dunnett’s multiple comparison test was performed, as appropriate, to compare the differently MSN-treated cells among themselves or with untreated time-matched controls at respective time points. In all cases, a two-sided *p* < 0.05 was considered statistically significant.

## 5. Conclusions

As the demand of usage of MSNs and their functionalized counterparts grows in the biomedical field, there is a need to study their potential adverse effects, considering not only the cytotoxicity, as a broadly studied parameter, but also the genotoxic and carcinogenic risk. Further, in addition to short-term exposures, the use of long-term exposure scenarios is essential in studies evaluating agents used directly in humans. In our work, we performed a wide range of assays which include the acute and long-term exposure of bare and functionalized MSNs and the detection of cytotoxicity, genotoxicity, and oxidative DNA damage, as well different biomarkers of cell transformation including migration, invasion, and secretome changes.

Our work seems to indicate that functionalized MSNs with PEG and GAL exerted some adverse effects regarding cell transforming ability, such as migration and invasion, in human lung cells chronically exposed to sub-lethal doses. Nevertheless, these exposures were barely effective in terms of cytotoxicity, oxidative stress, and genotoxicity. Because the adverse effects found are moderately low, and lower than those provoked by some approved drugs in clinics, MSNs could probably be used nanocarriers. On the other hand, the potential accumulation of MSNs in the organism is still controversial and, despite the long-term exposure approach in the current study, this approach might not fit with the in vivo physiological conditions. Thus, more studies with ‘realistic’ exposure conditions are needed to achieve a holistic view of the risk posed by MSNs in humans.

## Figures and Tables

**Table 1:** Characterization data of MSNs and gated MSNs under TEM, Zetasizer, and thermogravimetric analysis.

	MSNs	MSNs-PEG	MSNs-GAL
Size (nm) (TEM)	81.7 ± 11.2	82.4 ± 10.5	80.9 ± 14.0
Size (nm) (DLS)	148	245	300
PDI (width of the distribution) (DLS)	0.242	0.307	0.215
ζ Potential (mV)	−31.7 ± 5.6	−23.1 ± 6.2	−12.3 ± 4.37
Organic content (%)	–	23.1	18.2

## Data Availability

The data presented in this study are available online within this article or in the Supplementary Materials.

## Supplementary Materials

The following supporting information can be downloaded at https://www.mdpi.com/article/10.3390/ijms242216158/s1.
