# Supplementary material for: Toxicological Profiling and Long-Term Effects of Bare, PEGylated- and Galacto-Oligosaccharide-Functionalized Mesoporous Silica Nanoparticles
Source: Int J Mol Sci. 2023 Nov 10;24(22):16158. doi: 10.3390/ijms242216158 (PMC10671840; doi:10.3390/ijms242216158)
Supplement: Supplementary file 1 [file ijms-24-16158-s001.zip › ijms-2692690-supplementary.pdf]

SUPPLEMENTARY INFORMATION

Toxicological profiling and long-term effects of bare, PEGylated-and galacto-oligosaccharide-functionalized mesoporous silica nanoparticles

Irene Barguilla[1,#], Vicente Candela-Noguera[2,3#], Patrick Oliver[1], Balasubramanyam Annangi[1], Paula Díez[2,3,4,5], Elena Aznar[2,3,4,5,§], Ramón Martínez-Máñez[2,3,4,5], Ricard Marcos[1], Alba Hernández[1,§], M. Dolores Marcos[2,3,4,5]

[1]*Grup de Mutagènesi, Departament de Genètica i de Microbiologia, Facultat de Biociències, Universitat Autònoma de Barcelona, Cerdanyola del Vallès, Spain.*

[2]*Instituto Interuniversitario de Investigación de Reconocimiento Molecular y Desarrollo Tecnológico, Universitat Politècnica de València, Universitat de València, Valencia, Spain.*

[3]*Unidad Mixta UPV-CIPF de Investigación en Mecanismos de Enfermedades y Nanomedicina. Universitat Politècnica de València, Centro de Investigación Príncipe Felipe, Valencia, Spain.*

[4]*Unidad Mixta de Investigación en Nanomedicina y Sensores. Universitat Politècnica de València, Instituto de Investigación Sanitaria La Fe (IISLAFE), Valencia, Spain.*

[5]*CIBER de Bioingeniería, Biomateriales y Nanomedicina (CIBER-BBN), Instituto de Salud Carlos III.*

[§]Corresponding authors at:

Grup de Mutagènesi, Departament de Genètica i de Microbiologia, Universitat Autònoma de Barcelona, Edifici Cn, Campus de Bellaterra, 08193 Cerdanyola del Vallès (Barcelona), Spain.

E-mail: alba.hernandez@uab.cat (A. Hernández)

Instituto Interuniversitario de Investigación de Reconocimiento Molecular y Desarrollo Tecnológico, Universitat Politècnica de València, Universitat de València, Camino de Vera s/n, 46022, Valencia, Spain

E-mail: elazgi@upvnet.upv.es (E. Aznar)

[#] These authors have contributed equally to this work

CONTENTS

Methods

Release assays

Figures

Figure S1. Characterization of structure and mesoporosity of bare MSNs under PXRD and N2 adsorption−desorption isotherms.

Figure S2. Representative TEM images for bare MSNs, MSNs-PEG and MSNs-GAL.

Figure S3. Dynamic light scattering (DLS) curves of bare MSNs, MSNs-PEG and MSNs-GAL.

Figure S4. Thermogravimetric assay (TGA) curves of bare MSNs, MSNs-PEG and MSNs-GAL.

Figure S5. Controlled release assays monitoring curves.

Methods

Release assays

Delivery experiments were carried out with the capped materials MSNs-PEG and MSNs-GAL. 1 mg of MSNs-PEG was dissolved in distilled water and divided into two Eppendorf tubes, to one of which was added GSH (final concentration of 10 mM). 1 mg of MSNs-GAL was dissolved in distilled water at pH 4.5 and divided into two Eppendorf tubes, to one of which was added βGAL (final concentration of 3.5 mg/mL). The suspensions were then stirred, and some aliquots were separated and centrifuged at different times. Dye delivery was monitored through the emission band of SafO or RhoB, centered at 585 nm (λ_{exc} = 520 nm) or 575 nm (λ_{exc} = 555 nm), respectively.

Figures

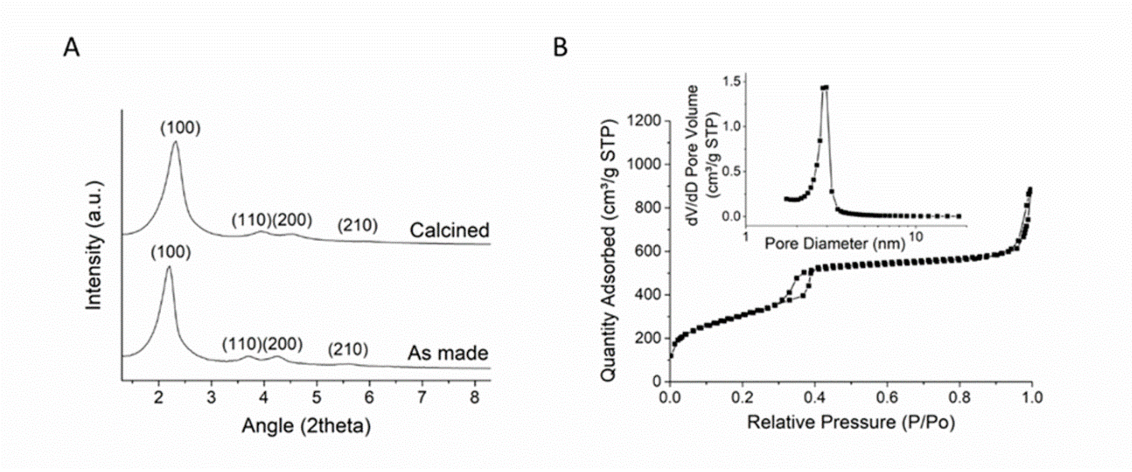

Figure S1. Characterization of structure and mesoporosity of bare MSNs under PXRD and N2 adsorption−desorption isotherms. (A) Diffractograms for bare MSNs, both as

made and calcined samples. (B) N2 adsorption−desorption isotherms for bare MSNs. Inset: pore-size distribution.

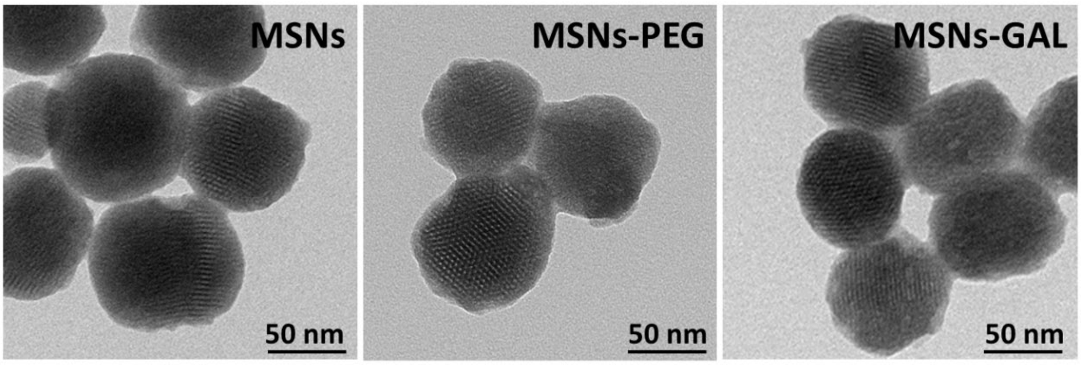

Figure S2. Representative TEM images for bare MSNs, MSNs-PEG and MSNs-GAL.

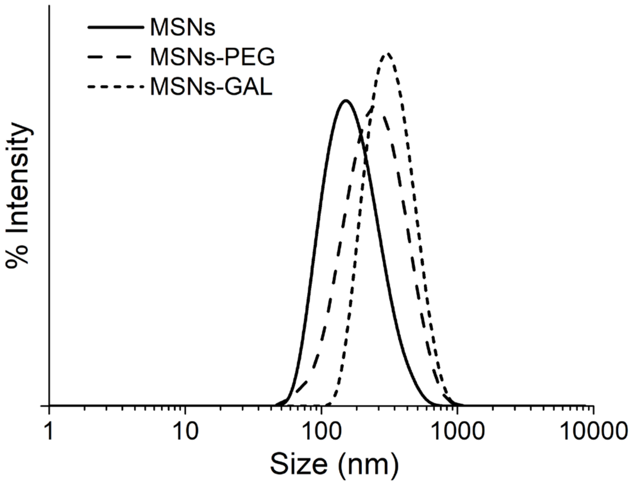

Figure S3. Dynamic light scattering (DLS) curves of bare MSNs, MSNs-PEG and MSNs-GAL.

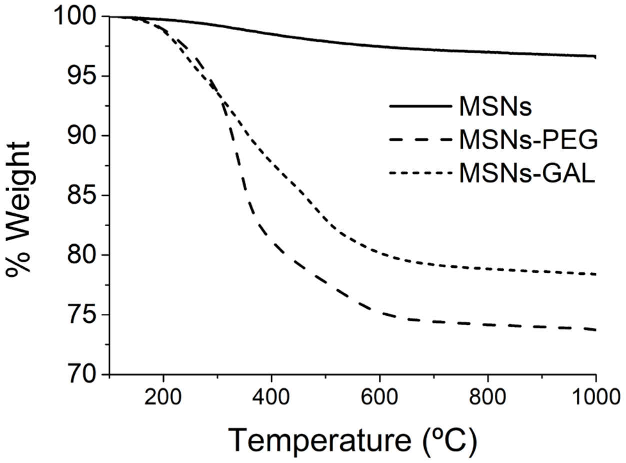

Figure S4. Thermogravimetric assay (TGA) curves of bare MSNs, MSNs-PEG and MSNs-GAL.

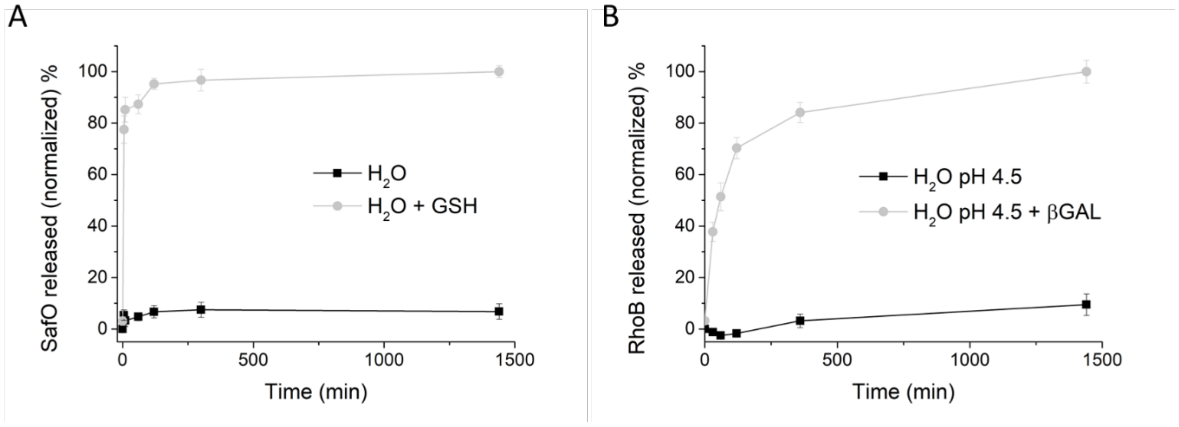

Figure S5. Controlled release assays monitoring curves.
